# Cloud-Based Automated Clinical Decision Support System for Detection and Diagnosis of Lung Cancer in Chest CT

**DOI:** 10.1109/JTEHM.2019.2955458

**Published:** 2019-12-04

**Authors:** Anum Masood, Po Yang, Bin Sheng, Huating Li, Ping Li, Jing Qin, Vitaveska Lanfranchi, Jinman Kim, David Dagan Feng

**Affiliations:** 1 Department of Computer Science and EngineeringShanghai Jiao Tong University 12474 Shanghai200240China; 2 Department of Computer ScienceUniversity of Sheffield 7315 SheffieldS1 4DPU.K.; 3 Shanghai Jiao Tong University Affiliated Sixth People’s HospitalShanghai200233China; 4 Department of ComputingThe Hong Kong Polytechnic University 26680 Hong Kong; 5 Centre for Smart Health, School of NursingThe Hong Kong Polytechnic University 26680 Hong Kong; 6 Biomedical and Multimedia Information Technology Research Group, School of Information TechnologiesThe University of Sydney 4334 SydneyNSW2006Australia

**Keywords:** Computer-aided diagnosis, nodule detection, cloud computing, computed tomography, lung cancer

## Abstract

Lung cancer is a major cause for cancer-related deaths. The detection of pulmonary cancer in the early stages can highly increase survival rate. Manual delineation of lung nodules by radiologists is a tedious task. We developed a novel computer-aided decision support system for lung nodule detection based on a 3D Deep Convolutional Neural Network (3DDCNN) for assisting the radiologists. Our decision support system provides a second opinion to the radiologists in lung cancer diagnostic decision making. In order to leverage 3-dimensional information from Computed Tomography (CT) scans, we applied median intensity projection and multi-Region Proposal Network (mRPN) for automatic selection of potential region-of-interests. Our Computer Aided Diagnosis (CAD) system has been trained and validated using LUNA16, ANODE09, and LIDC-IDR datasets; the experiments demonstrate the superior performance of our system, attaining sensitivity, specificity, AUROC, accuracy, of 98.4%, 92%, 96% and 98.51% with 2.1 FPs per scan. We integrated cloud computing, trained and validated our Cloud-Based 3DDCNN on the datasets provided by Shanghai Sixth People’s Hospital, as well as LUNA16, ANODE09, and LIDC-IDR. Our system outperformed the state-of-the-art systems and obtained an impressive 98.7% sensitivity at 1.97 FPs per scan. This shows the potentials of deep learning, in combination with cloud computing, for accurate and efficient lung nodule detection via CT imaging, which could help doctors and radiologists in treating lung cancer patients.

## Graphical abstract

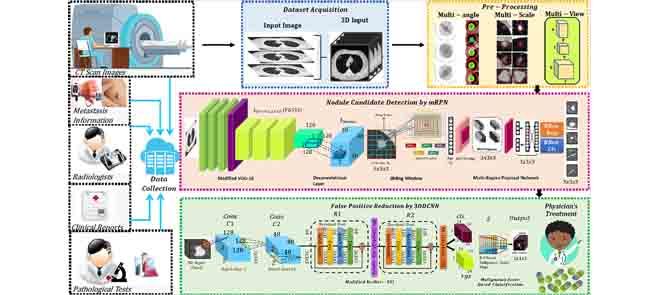

## I. Introduction

**FIGURE 1.**
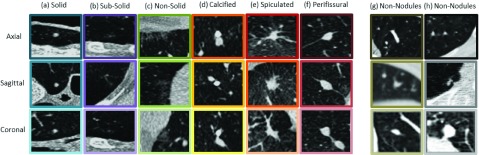
Nodules and non-nodules in coronal, sagittal and axial view (nodules/non-nodules positioned at the center of the box ($40\times 40$ mm). Left images set are various types of nodules: (a) Solid (b) Sub-Solid (c) Non-Solid (d) Calcified (e) Spiculated (f) Perifissural while right images set are non-nodules.

Among different types of cancer, pulmonary cancer also refer to as lung cancer is considered to be one of the most deadly cancers. In 2018, there were approximately 2.2 million new pulmonary cancer cases and about 1.8 million deaths in U.S. within a year. Pulmonary cancer is an uncontrollable abnormal lung cells growth, referred to as nodules, whose detection in early stages is highly crucial to the effective control of disease progression and thus potentially increase the survival rate of the patient. Commonly used manual lung nodule delineation by radiologists on high-resolution and high-quality chest Computed Tomography (CT) is complex, time consuming and extremely tedious [1]. Automation of pulmonary nodule detection with effective and efficient Computer-Assisted Diagnosis (CAD) tools facilitates radiologists in fast diagnosis and improves the diagnostic confidence. Among these approaches, one key challenge in CAD systems for lung cancer is dealing with the morphological variations in the nodules in CT images. Usually such variations are evident in images with different image modalities such as Magnetic Resonance Imaging (MRI), Positron Emission Tomography (PET), X-ray or CT but in case of lung nodules numerous morphological variations are present even if the same image modality is used [2], [3]. Fig. 1 shows various nodules and non-nodules examples which depict the variety of morphological features, resulting in complexity in data used for nodule detection and diagnosis systems. Recently, deep learning methods [4], [5] have merged both the hand-designed feature extraction process and nodules classification process into a combined automated training process. Deep learning techniques have demonstrated great performance (i.e. reduced number of False Positive (FP) results) when compared with typical results reported by deploying traditional segmentation techniques [6], [7]. This paper presents a novel computer-aided decision support system for lung nodule detection. The contributions of this paper are threefold.

• A novel automated clinical decision support system for lung detection based on a 3D Deep Convolutional Neural Network (3DDCNN) architecture. In order to leverage 3-dimensional information from CT scans, we applied novel median intensity projection and introduced a novel multi-Region Proposal Network (mRPN) in our architecture for automatic selection of potential region-of-interest.
• To further improve the efficiency and performance of our proposed model, we integrated cloud computing into our CAD system. Proposed computer-aided decision support system is used for nodule detection and for assistance of radiologists in clinical diagnosis at Shanghai Sixth Peoples Hospital.
• A comprehensive experimental evaluation of our CAD system done on four different datasets with varying CT imaging parameters with existing state-of-the-art CAD systems for lung cancer detection demonstrated that our system outperformed the existing systems and obtained an impressive 98.7% sensitivity at 1.97 FPs per scan.

The rest of the paper is organized as follows. Section 2 briefly introduces the related work, detailed description of our method is presented in Section 3. In Section 4, we discussed the experimental results on different datasets. Our paper is concluded with relevant future work in Section 5.

## II. Related Work

CAD system is one of the most common means to improve the accuracy of cancer diagnosis done by the radiologists and decrease the time required for interpretation of the CT images. CAD systems are further categorised as: Computer Aided Detection (CADe) systems and Computer Aided Diagnosis (CADx) systems. CADe systems assist in finding the locality of nodules in CT images acquired from different imaging modalities while on the other hand the CADx systems characterize and classify these detected lesions as malignant or benign tumors. In general, a CAD system designed for the detection of pulmonary lesions (nodules) has two steps namely candidate nodule detection and FP Elimination. Firstly, the Regions Of Interest (ROIs) are selected in the input CT image, then the lung nodule candidates are extracted. Teramoto and Fujita [8] used Active Contour Model (ACM) filter for enhancement of contrast then used thresholding of the resultant images for the screening of candidate nodules. Supervised learning methods namely linear discriminant analysis (LDA), gray-scale distance transform, clustering ($k$-means clustering), connected component analysis, and patient-specific priori model have been used in conventional approaches [9]. FP reduction step classifies the lung nodules and non-nodules using machine learning techniques. The main objective is to eliminate the FP results which are considered as candidate in the previous step. Hierarchical Vector Quantization (HVQ), Rule-based filter, LDA, Artificial Neural Networks (ANN), and Support Vector Machine (SVM) are few supervised reduction methods which are used for FP reduction. Random Forest (RF) is reported to surpass SVM in FPs reduction in lung CAD system. Regression tree-based classifiers have shown efficient discrimination ability in reduction of FPs for improved detection results [10]. Spatial and metabolic features in combination with SVM [8] are other approaches used for FPs elminiation.

In the past few years, researchers have presented deep learning based CAD systems for cancer detection with promising results [11]. Convolutional Neural Network (CNN) framework is used for FPs reduction [12]. Nodules were accurately classified by using the fully connected layers (Fc) of CNN integrated with SVM classifier in [13]. Shen *et al.* [14] proposed a Multi-Crop CNN (MC-CNN) comprising of training by cropped convolutional feature maps and max-pooling layers recursively. Multi-View CNN proposed by Setio *et al.* [15] combines three candidate detectors each for sub-solid, solid, and large nodule category and then utilizes a fusion method to classify the input CT image. A 3-dimensional Fully Convolutional Network (FCN) based on Volumes Of Interest (VOI) was employed for classification [16]. This proposed work produces a score map with respect to the input VOI in single pass which is used for training of CNN used for classification. Deep learning based models have also been proposed for the candidate nodule detection [17], [18]. Multi-scale Laplace of Gaussian (LoG) filters and shape priors based multi-scale 3D-CNN model is proposed in [19]. In the past few years both CADe and CADx systems have been researched independently. CADe’s major shortcoming for detecting lung cancer is their lack of ability to characterize them. CADe systems assist the radiologists in detection of lung nodules but do not provide detailed radiological characteristics of the lesion, consequently missing the information which is crucial for radiologists, while on the other hand CADx systems do not automatically identify lesions thus they do not possess high automation levels, making it not suitable for clinical use. Therefore, a new and advanced CAD system is needed, that incorporates the benefits of detection from CADe and diagnosis from CADx into a single system for better performance. The CADx systems performance evaluation is conducted in terms of computational efficiency, accuracy, sensitivity and specificity.

## III. Materials and Methods

### A. Training Datasets

For the training of our proposed method for nodule detection, we used LUng Nodule Analysis (LUNA16) dataset [20] which comprises of 888 annotated CT scans. In these CT scans, four radiologists marked the lesions as nodule $ < 3mm$, nodule $\geq 3mm$, or non-nodule in a two-phase annotation process. We used 55 CT-scans from ANODE09 dataset [21], among which only 5 have annotations done by three radiologists containing 39 nodules and 31 non-nodules. We used the remaining 50 as testing datasets which contained 433 non-nodules and 207 nodules along with LIDC-IDR dataset [22] to validate the nodule detection and classification of our proposed method. Since we were using two heterogeneous datasets having varied image resolution therefore we resampled CT scans by the help of spline interpolation by 0.5mm per voxel along $x$, $y$ and $z$-axis to have constant resolution and we further reconstructed all the images by sharp kernel (Siemens B50 kernel).

### B. Data Augmentation

CNN models have a tendency to overfit data in case of limited labeled training dataset [12], therefore to ensure that the training of our model does not overfit, we trained our model with data-augmented training dataset. Since benign nodules are more in number as compared to malignant nodules, we choose to augment the malignant training samples by cropping, duplicating, random translation within the range of [1, 0, or −1 pixels in each dimensions (3D)] voxels, flipping, scaling, swapping in three dimensions axes and then rotating on the angle of [0, 90, 180, 270 degree] in training dataset. Specifically, among input batch, random translation as well as rotation are performed for up-sampling and down-sampling. These data-augmentation methods assisted our model in capturing nodule attributes invariant to image-level affine transformations.

### C. Pre-Processing

#### 1) Multi-Scale ROI Patches

**FIGURE 2.**
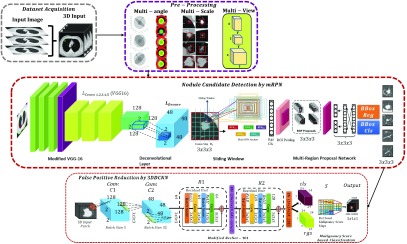
Our CAD system comprises of four stages: data acquisition (2D CT scan image to generate MIP projected images), pre-processing (multi-angle, multi-scale and multi-view), candidate screening (mRPN for nodule detection), false positive reduction using 3DDCNN.

Multi-scale ROI patches were generated by zooming in or out of the CT image in coronal, axial and sagittal views (see Fig. 1). The motivation for the multi-scale ROI patches comes from the real life scenario when the radiologists detects cancer patterns in a patient’s CT-scans. In this scenario, the suspected regions were explored on pixel level in the follow-up check-up thus making these regions more scrutinized than the rest. Therefore, the training dataset comprising of nodules was used in different multi-scale patches as shown in Fig. 2. Using multi-scale ROI patches also upsampled the labeled dataset.

#### 2) Multi-Angle ROI Patches

Multi-angle ROI patches were generated by rotation of obtained multi-scale patches by small angle $\theta $ in orthogonal coordinate systems to obtain labeled data. The deep learning based methods can easily process these multi-scale and multi-view ROI patched to their properties such as shift invariance [23].

#### 3) Multi-View Combination

In a two dimensional lung CT scans, most of the FPs are caused by the trachea and the blood vessels having tubular spatial structure. To reduce the noise caused by trachea and the blood vessels, we used a three dimensional bilateral smoothing filter $S(x, y, z)$ for the pre-processing of CT-scans $C(x,y,z)$.

\begin{equation*} S_{i}(x, y, z)=n_{c}^{-1}\sum \limits _{(\theta,\xi,\eta)\in \Omega _{w}}C(\theta,\xi,\eta)w_{c}(\theta,\xi,\eta; x, y, z)\tag{1}\end{equation*}

where $S_{i}(x, y, z)$ is the output smoothed image, $n_{c}^{-1}$ is the normalization coefficient and $\Omega _{w}$ means the filter window $w_{c}(\theta,\xi,\eta; x, y, z)$ denotes the weight coefficient which can be described as:

\begin{align*}&\hspace {-2pc}w_{c}(\theta,\xi,\eta; x, y, z) \\&=w_{G}((\theta,\xi,\eta),(x, y, z))\cdot w_{E}(C_{\theta,\xi,\eta }, C_{x, y, z})\tag{2}\end{align*}

where $w_{G}((\theta,\xi,\eta),(x, y, z))$ expressed geometric similarity whereas $w_{E}(C_{\theta,\xi,\eta }, C_{x, y, z})$ expressed energy similarity. For removal of the effect of trachea and the blood vessels meanwhile enhancing the ROIs, three dimensional isotropic Gauss function was utilized. Thus, the weight coefficient can be described as follows:

\begin{align*}&\hspace {-2pc}w_{c}(\theta,\eta,\xi; x, y, z)=\big[-\frac {1}{n}\left({\frac {\theta -x}{\sigma _{g}}}\right)^{2}+\left({\frac {\xi -y}{\sigma _{g}}}\right)^{2} \\&\qquad \qquad \qquad +\left({\frac {\eta -z}{ \sigma _{g}}}\right)^{2}\big)\big].\left[{-\frac {(C_{\theta,\xi,\eta }-C_{x, y, z})^{2}}{2\sigma _{E}^{2}}}\right]\tag{3}\end{align*}

where $\left.\left[-\frac{1}{n}\left(\frac{\theta-x}{\sigma_{g}}\right)^{2}+\left(\frac{\xi-y}{\sigma_{g}}\right)^{2}+\left(\frac{\eta-z}{\sigma_{g}}\right)^{2}\right)\right]$ represents the geometric similarity, $\left[{-\frac {(C_{\theta,\xi,\eta }-C_{x, y, z})^{2}}{2\sigma _{E}^{2}}}\right]$ represents the energy similarity and $\sigma _{E}$ and $\sigma _{g}$ represents the standard deviation $(\sigma)$ of gray-level energy values and Gauss function. The terms $C(\theta,\xi,\eta)$ and $C(x,y,z)$ are related to CT scan gray-level energy values therefore they are referred to as energy similarity whereas $\left(\frac{\theta-x}{\sigma_{g}}\right)^{2},\left(\frac{\xi-y}{\sigma_{g}}\right)^{2},\left(\frac{\eta-z}{\sigma_{g}}\right)^{2}$ represent geometric similarity since these terms are related to the spatial structure of the anatomical structures. We used $-\frac {1}{n}$ with the geometric similarity where $n$ represents the independent planes, in case of energy similarities there is no clear gray level boundaries therefore we used − with the energy similarity. The distance between $(\theta,\xi,\eta)$ and $(x, y, z)$ is the distance between the spatial nodule localization and the gray level energy difference between nodule and other anatomical structure. This similarity metric is the first decision making step to reduce the undesirable feature redundancies which tend to cause false positive results. This metric ensures that the nodule class similarity is maximized on the other hand the non-nodule to nodule class similarity is minimized. Traditionally the Gaussian function refers to mean and co-variance matrix but when the number of independent parameters increase as the number of dimension increase then the multi-dimensional isotropic Gaussian distribution is considered where the variance of each dimensional is the same. In our case, we have three dimensional MIP projected images with multi-view combination therefore the 3D isotropic Gauss function was used. Afterwards an enhancement filter is used which suppresses the spatial tubular structure but enhances the nodule structures. Although commonly the classification phase has a problem when the input channels have multiple color channels, yet in our CT-scans dataset we only have gray-level images. CT input training images are 2-D whereas the locality of the nodule is presented by $z$-axis leveraging the inter-slice dependencies through memory units are three dimensional. Thus, to combine the multi-views of three dimensions of CT-scans, each voxel of the 3-D CT scans input was processed by the dot-enhancement filter which is inspired by the $3\times 3$ Hessian matrix. We define $\theta $ as the image projected by Maximum Intensity Projection (MIP). With input image patch $I$, $\vartheta $ for three dimensions can be presented as:

\begin{align*} \vartheta (y_{i},z_{i})=&\mathop {med}\limits _{x_{i}} I(x_{i},y_{i},z_{i}) \\ \vartheta (x_{i},z_{i})=&\mathop {med}\limits _{y_{i}} I(x_{i},y_{i},z_{i}) \\ \vartheta (x_{i},y_{i})=&\mathop {med}\limits _{z_{i}} I(x_{i},y_{i},z_{i})\tag{4}\end{align*}

where $med$ denotes median operator.Different views can provide different plane information, while patches with combination of different dimensions can provide the space distribution of tumor tissues. In order to construct input image sets with three channels, we connect three MIP projected images together: $\vartheta = [\vartheta (y_{i},z_{i}), \vartheta (x_{i},z_{i}), \vartheta (x_{i},y_{i})]$.

### D. Proposed Model Architecture

Our proposed architecture uses the basic framework of the Faster R-CNN [24]. Candidate detection is done by the proposed mRPN while the FP reduction is done by novel 3D DCNN.

#### 1) Candidate Detection by mRPN

**FIGURE 3.**
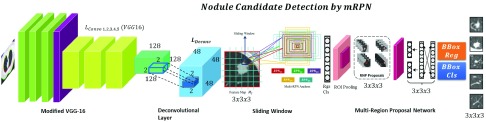
Overview of the Proposed mRPN architecture for Nodule Candidate Detection using modified VGG-16 baseline for $L_{convo (1,2,3,4,5)} integrated with $ deconvolutional layer $L_{deconv}$, feature map $M_{f}$, multi-RPN anchor with Classification (cls) and Regression (rgs) layers.

To use the training ability of CNN model for lung cancer, the model should be both end-to-end and trained frequently trainable for ROI detection and classification such as, Faster R-CNN [24]. The challenge for Faster R-CNN [24] in case of pulmonary lesions detection is the diversity in the nodule size and limited labeled dataset. We proposed a novel method, mRPN that has enhanced the feature extraction process (multi-resolution) and uses varied window size for region proposal selection from ROIs. For efficient ROI selection, these ROI extracted from multiple RPNs are merged in an additional layer as shown in Fig. 3. Our proposed novel model mRPN, which is based on based on VGG-16 Net model proposed by [25], takes a CT image (of any size) as input and generates a set of rectangular region proposals, each outputs an objectness score. Our network Multi Region Proposal Network (mRPN) hyper-parameters of all layers from conv1 to conv5 are similar to the VGG16 model. The original VGG-16 model [26] comprises of multiple max pooling layers, which inevitably reduces the image size but simultaneously distort the relatively small sized malignant nodule. We used a small network $N_{s}$ to slide through the activation (feature) map output $M_{out}$ by the final added deconvolutional layer $deconv_{L}$. We proposed a deconvolutional layer $deconv_{L}$ [27], 4 kernel size and 4 stride size, to be added after the last feature extracting layer. The deconvolutional layer $deconv_{L}$ (or more commonly known as transposed convolutional layer) upsampled the features learned from the input and the feature map $M_{f}$. This $deconv_{L}$ upsample the feature maps that are derived from the downsampling stack to generate $M_{out}$, while ensuring that both the output $M_{out}$ and input $M_{in}$ have the same size. Traditionally, R-CNN depends on the skip connection linked with the deconvolution layer on the upsampling for generating initial results but the deconvolution layer is unable to recover the small-sized objects such as nodules, which are lost after the downsampling. Therefore, they cannot accurately detect small-sized nodules. In our proposed method, we used $deconv_{L}$ which ensured the recovery of any loss of small objects such as lung nodules in the downsampling process.

**FIGURE 4.**
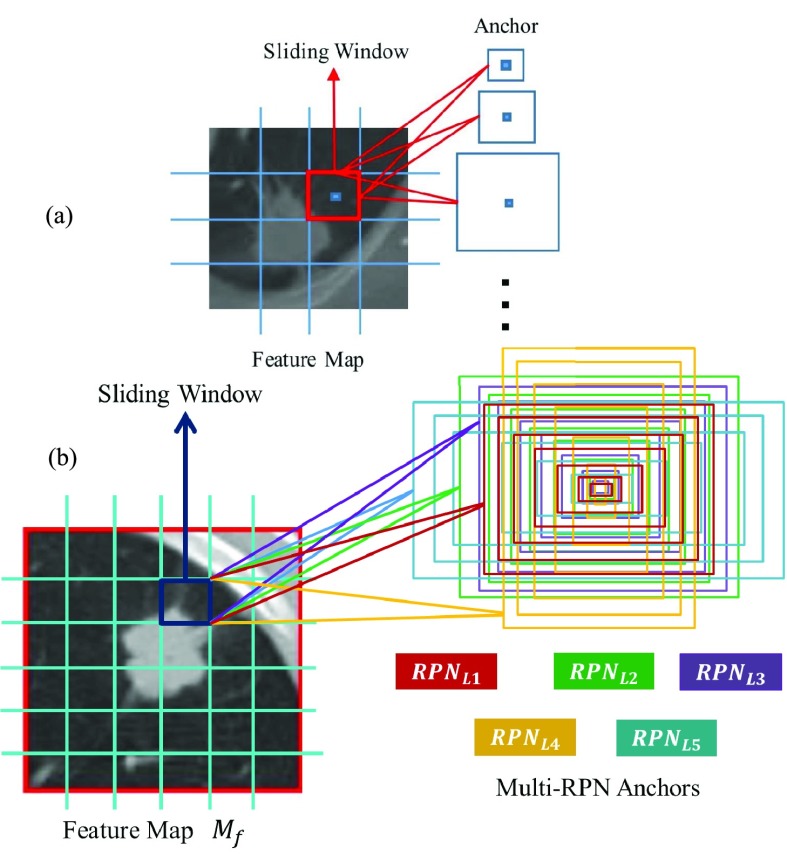
(a) Existing anchor in region proposal network. (b) Proposed anchor in region proposal network.

The proposed anchor in RPN [24] is shown in Fig. 4. We explored large number of nodule boundary varying in sizes, and generated seven different sizes of reference bounding boxes which are centered at each sliding spatial window $w$, in order to contain nodules of different malignant level, we choose anchor sizes of $4 \times 4$, $8 \times 8$, $12 \times 12$, $16 \times 16$, $20 \times 20$, $26 \times 26$, and $32 \times 32$. These 7 anchors are divided into RPN levels targeting nodules diameter $\tau $ ranging from 3mm to 35mm in different aspect ratios and different sizes. These different RPN levels work in a cascade manner and overall increase the efficiency of the proposed model performance as shown in Fig. 4(b). Each of these have a $1\times 1$ convolutional layer with about 28 units for the Bounding Box Regression (BBReg) and $1\times 1$ convolutional layer having 14 units for the Bounding Box Classification (BBcls). The BBReg with 28 units gives an output of (H,W,28) size. This output is used for providing four regression coefficients for each of the seven anchors for each point in the feature map $(H\times W)$. These four Regression (Rgs) coefficients are further used to enhance the coordinates of the anchors that is comprised of nodules. On the other hand the BBcls with 14 units provide an output (H,W,14) which is used to obtain classification (cls) probabilities for each of the point of feature map (H,W) whether it contains a nodule within these seven anchors at the given point or not.

Nodules detection carried out using the different levels of RPN results in improvement of the nodule detection since both diameter and volume are considered. Volumetric values (3D input) are in correlation with the diameter values (2D input), therefore the combination of both volume and diameter provides divergence for non-nodules.

#### 2) False Positive Reduction by 3DDCNN

**FIGURE 5.**
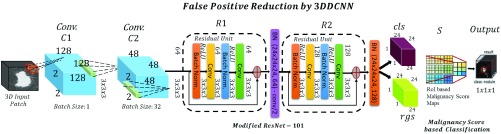
Our 3DDCNN model for False Positive Reduction comprising of Convolutional Layer $conv C_{1}$ and $conv C_{2}$, Residual Unit $R_{1} + BN$, $R_{2} + BN$, Classification (cls) and Regression (rgs) layers, Scoring Layer $S$ and Output Layer generating the malignancy score based classification as nodule (malignant or benign) or non-nodule.

False Positive reduction is carried out using by novel 3DDCNN which is inspired by the ResNet-101 network [28]. The 3DDCNN replaces 2D with 3D convolution. This is used to predict the presence of nodules or to classify if a nodule exists on the basis of the malignancy value. VGG16 Net model is the basic layout of the 3DDCNN [26] having 100 convo layers with stride:2 where filters of size$3\times 3\times 3$ used. Furthermore, this architecture network was improved by adding connection for shortcuts converting it into its comparable counterpart residual network as shown in Fig. 5.

**FIGURE 6.**
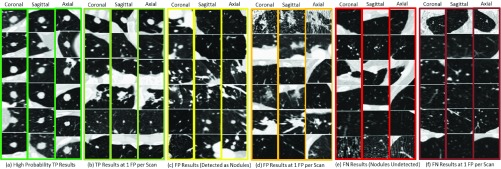
True Positive (TP), False Positive (FP), False Negative (FN) results of our proposed CAD system (nodules positioned at the center of $40\times 40mm$ Patch).

In case of 3DDCNN, the feature map is defined as:

\begin{equation*} \omega =\sum _{I}({x_{i}}, {y_{i}},{z_{i}})\tag{5}\end{equation*}

The input set was 3D CT image therefore $\omega $ corresponds to the 3D position (e.g. ${x_{i}}, {y_{i}},{z_{i}}$).

\begin{equation*} \nu =\frac {\tau +\upsilon +\kappa }{n_{RPN}}\tag{6}\end{equation*}

where $\tau $ is the diameter of the detected nodule, $\upsilon $ is the volume, $\kappa $ is the malignancy score which is obtained by using the algorithm proposed in [11] and $n_{RPN}$ is the total number of RPN levels used. For our model, value of $n_{RPN}$ is 7.

\begin{equation*} M_{f}=({\omega +\nu +\varphi }) \times W_{I} \times H_{I} \times \frac {D_{I}}{\mu }\tag{7}\end{equation*}

where $\varphi $ is the confidence score, $W_{I}\times H_{I} \times {D_{I}}$ gives the input width, height and depth of $128\times 128\times 128$, respectively. The confidence score represents the probability value of an anchor to contain a nodule. In our model we used the confidence score as a threshold for determining whether the detected nodule is a FP or a True Positive (TP) result. We set the threshold for confidence score at various levels and obtained 95% confidence interval. Due to memory limitation, the $\frac {D_{I}}{\mu }$ is used where the spatial scale factor presented by $\mu $ is 3. The 3DDCNN is designed to acquire spatial scale $\mu =3$ between the input $M_{in}$ and the output $M_{out}$ space. The first convolution layer (size of kernel: $7\times 7\times 7$, stride:2) of 3DDCNN is applied on the input set on bidirection. In 3DDCNN, we have 3D convolution layer (kernel size: $7\times 7\times 7$, stride:2), followed by BN layer (batch normalization) and ReLu (rectified linear unit) activation. In order to keep low ratio among the feature map output $M_{out}$ and the feature map input $M_{in}$, we proposed to use only two ResNet blocks having a single residual connection (kernel size: $3\times 3\times 3$). One of the ResNet block has feature stride:1 while the other has stride:2. In case of similar dimensions for both the output and input, the identity alternatives can be directly used:

\begin{equation*} q = \mathbb {F}(p, \{ W_{i}\}) + p\tag{8}\end{equation*}

where $p$ and $q$ denotes input and output sets respectively, that are fed into and considered by each network layer. Residual mapping learning function is defined as $\mathbb {F}(p, \{ W_{i}\})$. The 3DDCNN architecture modification is done by using convolutional layer for generating $M_{f}$, thus, the learnable weights are computed using convolutional layers which are shared on the image level.

According to radiologist annotations, we add a $k \times k(5 + 1)$-channel convolutional layer ($4 \times 4$ in this paper) as the output layer to generate position sensitive score maps. Note that 5 represents five malignant level of lung nodule, 1 represents non-nodule, and we divided RPN levels proposed ROI into $4 \times 4$ grid cell. Specifically, for $32 \times 32$ proposed rectangular, each divided grid has the size of $8 \times 8$. Therefore $4 \times 4 \times 7$ score maps will be generated, and we use the average pooling operation to calculate the relevance score to 7 categories for each split bin:

\begin{equation*} \zeta _{c}(w,h \mid \phi) = \sum _{(a,b) \in bin(w,h)} z_{w, h, c}(a + a_{0}, b + b_{0} \mid \phi) / n\tag{9}\end{equation*}

where $\phi $ denotes parameters of the network, $\zeta _{c}(w,h \mid \phi)$ is the relevance score of $(w, h)$th bin to malignant category $c$, $z_{w,h,c}$ is the score map generated by last convolutional layer, $(a_{0},b_{0})$ is the top-left corner of ROI, and $n$ denotes the total pixel number in the bin. With $4 \times 4 \times 7$ relevance scores $\zeta $ being calculated, the scoring layer $S$ decide the malignancy level of the ROI by simply average voting and also apply cross-entropy evaluation for ranking ROI:

\begin{equation*} \zeta _{c}(\phi) = \sum _{w,h} \zeta _{c}(w,h \mid \phi)\tag{10}\end{equation*}

Here $\zeta _{c}(\phi)$ denotes the relevance score for ROI to class $c$, and $\xi _{c}(\phi)$ is the softmax response for class $c$.

### E. Training Process

In the training process with multiple RPNs providing the region proposals, RPN loss function is applied for each RPN and Fast R-CNN loss function in an iteration. Our loss function can be defined by merging box regression and the cross-entropy loss:

\begin{align*} \mathbb {L}_{t,\xi }=&-\log (\xi _{c^{*}}) + \frac {1}{N_{r}}\sum \mathbb \{L\}_{r}(t,t^{*}) \tag{11}\\ \mathbb {L}_{r}(t,t^{*})=&\begin{cases} 0.5(t-t^{*})^{2}, \{if \} \left |{ t - t^{*} }\right | < 1\\ \left |{ t - t^{*} }\right | - 0.5, \& \{otherwise\} \end{cases}\tag{12}\end{align*}

where the left part of the above equation denotes classification cross entropy loss [28], $N_{r}$ is the input number of Regression layer, $\mathbb {L}_{r}$ is similar to the bounding box regression loss as in [24], $t^{*}$ denotes ground truth values while $t$ denotes predicted values. $\xi _{c^{*}}$ represent Intersection-over-Union (IoU) between any two entities that is their overlap volume divided by their union volume. In our model, IoU is used to select the best anchor to acquire nodule feature with least transformation. If an anchor of $RPN_{Lx}$ has highest IoU, i.e. $IoU_{max}$ with regards to any of the $t^{*}$ or if $IoU>0.5$, then the said anchor is considered Positive $An^{P}$, whereas those anchors having $IoU < 0.02$ are considered Negative $An^{N}$ and anchors which are neither Positive nor Negative are irrelevant. A hard negative mining method was used to enhance the generalization. The classification imbalance was adjusted by normalizing the weights for classes (non-nodule, benign, malignant). For negative anchors, $An^{N}$ weight was the probability of nodule class while on the other hand the positive anchors $An^{P}$, weight was 1. Learning rate was initially set to 0.001 while the decay method for learning rate was done every 30 epochs, the learning rate was halved. The CAD system was trained for 300 epochs using the batch size (size:32). In order to leverage the gradient information from these selected batches, average gradient operation was performed on the $B$ samples and used as the input gradient estimation for Adam process to iteratively optimize the 3DDCNN. The experiment was conducted on Ubuntu 16.04.3 LTS with 4 processors, Intel(R) Xeon(R) CPU E5-2686 v4@2.3GHz and 64GB total memory space. Our model is trained on Tesla K80 with 12GB Memory. We used Intel Extended Caffe for implementation of 3DDCNN model.

### F. Cloud-Based 3DDCNN CAD System

In this paper, we have proposed a two stage computer-assisted decision support system for lung cancer detection. To further improve the performance of our proposed method, we integrated cloud computing (Infrastructure as a Service (IaaS) by providing Virtual Machines, and Software as a Service (SaaS) by giving our 3DDCNN model) into our CAD system. The first stage for our proposed CAD system is the training of 3DDCNN model for the nodule candidate screening. The second stage is the reduction of false positive results from the first stage in order to improve the overall diagnosis decision making by our CAD system as shown in Fig. 2. The final decision from the proposed model is provided to the radiologists to assist their diagnosis for lung cancer. The diagnosis decision by the proposed CAD system is sent to the doctors in real-time who determine the cancer stage. These physicians afterwards sent the regular check-up reports and treatment prescription to the patient. Treatment prescription and check-up reports are stored on the cloud storage for further data analysis and improvement of our CAD system. To efficiently identify the effectiveness of each of the prescribed treatment for specific lung cancer stage patient.

The proposed CAD system uses body area network (BAN) comprising of sensors attached to patients body to record physiological information and CT-scan for chest CT which are stored on the cloud storage and undergo pre-processing. Furthermore gateways are used to forward that data to storage cloud for further processing. We deployed 12 VMs, and 24 processing units in our dedicated cloud back-end. For each case the complete processing time is about 219 ± 25.47 seconds. HTCondor tool was used for real-time optimization and monitoring of computing resources, thus the radiologists and the physicians have updated responsive CAD system. 3DDCNN CAD system SaaS model was used to provide supportive decision support system for assistance of the radiologists whereas IaaS provided the GPU-acceleration, fast computation as well as storage resources. This model provides automatic support for on demand scalability of computing and storage resources. Moreover, Cloud-based 3DDCNN CAD system is more efficient and provides cost-effective solution since CAD results can be reviewed in real-time by multiple radiologists while a cloud back-end is taking care of computations. On the other hand, traditional stand-alone CAD systems have low performance and high computational cost with no feedback from multiple radiologists in real-time.

## IV. Experimental Results

In this research work, we used two phase validation. The first phase is nodule detection without classification as done by other researchers [7], [29]. The second phase combines the performance of independent detection with the classification results to provide the overall performance evaluation of the CAD system. For the nodule detection, we used Free-Response Receiver Operating Characteristic (FROC) [30] including average sensitivity and the number of FPs per scan (FPs/scan) which is the official evaluation metric for LUNA16, where detection is considered a true positive if the location lies within the radius of a nodule centre. The classification performance is evaluated by the area under the ROC curve (AUROC) which shows the performance of our proposed method on classification of nodules as nodules (malignant or benign) or non-nodules.

### A. Training

Our 3DDCNN model was trained for 300 epochs during each fold of cross-validation. After approximately training 100 epochs, the loss on the validation set became more stable. The result for each fold was selected to be the one with lowest malignancy prediction loss on the validation dataset. If nodule malignancy is $M < 3$, the nodule belongs to benign class, whereas if nodule has $M\geq 3$ then it is categorized as a malignant nodule. Since we obtained the results of detection results on three malignancy levels $M$, we used 10-fold cross-validation to merge the detection results of three levels.

### B. Nodule Detection

#### 1) Nodule Detection Using LUNA16 Dataset

**FIGURE 7.**
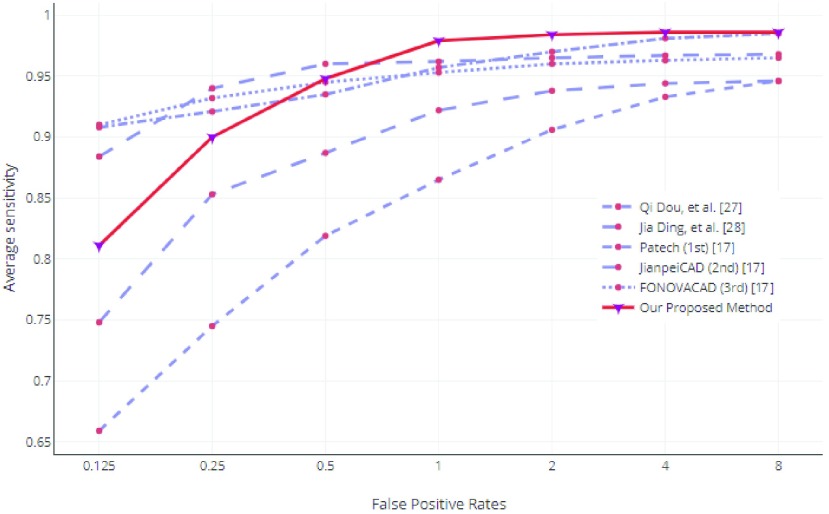
Performance comparison between our CAD system versus state-of-the-art CAD systems on LUNA16. We compared our results with Top three CAD systems of LUNA16 Challenge namely Patech, JianpeiCAD, FONOVACAD [20] and the two published state-of-the-art CAD systems based on LUNA16 dataset i.e. Dou *et al.* [31] and Ding *et al.* [32].

For evaluation of our proposed method’s nodule classification, we compared our results with the two state-of-the-art published methods, i.e. Dou *et al.* [31] and Ding *et al.* [32] along with the best three LUNA16 challenge [20] by calculating the average sensitivity over 7 FPs/scan [0.125, 0.25, 0.5, 1, 2, 4, 8 FPs/scan]. Our method demonstrated best performance for nodule detection sensitivity of 0.812, 0.901, 0.948, 0.978, 0.984, 0.9853, 0.9866 at respective FPs/scan, obtaining an average FROC score of 0.946 shown in Fig. 7.

### C. Nodule Detection and Classification

#### 1) LIDC-IDR and ANODE09 Dataset

We used a holdout validation set from LIDC dataset [20] and ANODE09 [21] to validate each model from 10-fold cross validation. For final results on LIDC, we retained 100% recall for validation sets, and reach 94.26% for nodules $ < 3mm$ at 2.9 FP/scan. Since it was not easy to process above 1000 test-sets in single step, we acquired result by cascading two FP elimination networks.

**FIGURE 8.**
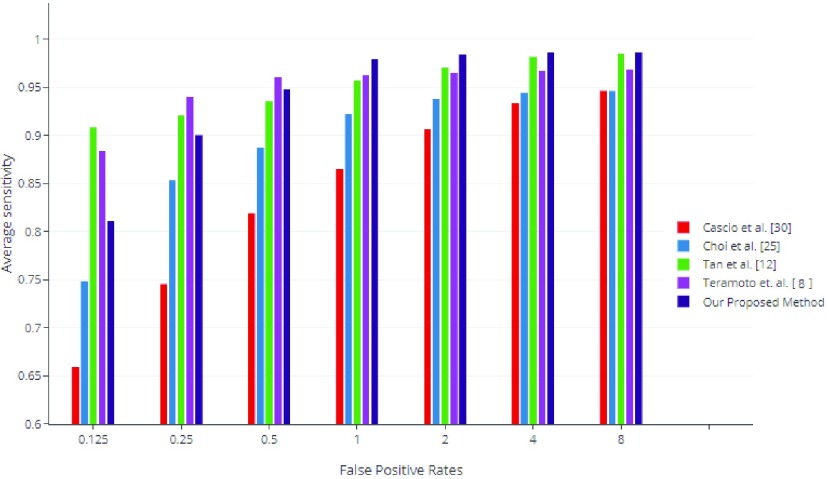
Performance comparison between our CAD system versus state-of-the-art CAD systems on LIDC-IDR dataset.

The effectiveness of 3DDCNN is verified by comparing with CNN [12], Autoencoder [10], Massive-feat [9], MC-CNN [14], MTANNs [33] and FCN [13]: the results are depicted in Table 1. The performance comparison between our proposed method for candidate nodules versus the state-of-the-art method in terms of sensitivity, specificity, AUROC and FP rate is shown in Fig. 8. Our proposed method improves 3.9% FROC on average over other state-of-the-art systems based on LIDC-IDR. The improvement on holdout test data validates our proposed method as an effective model to exploit potentially large amount of datasets which would not require further costly annotation by expert doctors and can be easily obtained from hospitals.

**TABLE 1:** Comparison of Various Classifiers’ Accuracy (%) on LIDC-IDR and ANODE09 Datasets

Classifiers	Accuracy(%)
CNN [12]	80.8
MTANNs [33]	88.6
FCN [13]	91.2
Autoencoder [10]	80.29
Massive-feat [9]	83.21
MC-CNN [14]	87.14
Our Proposed Method	98.51

#### 2) LIDC-IDR Dataset

**FIGURE 9.**
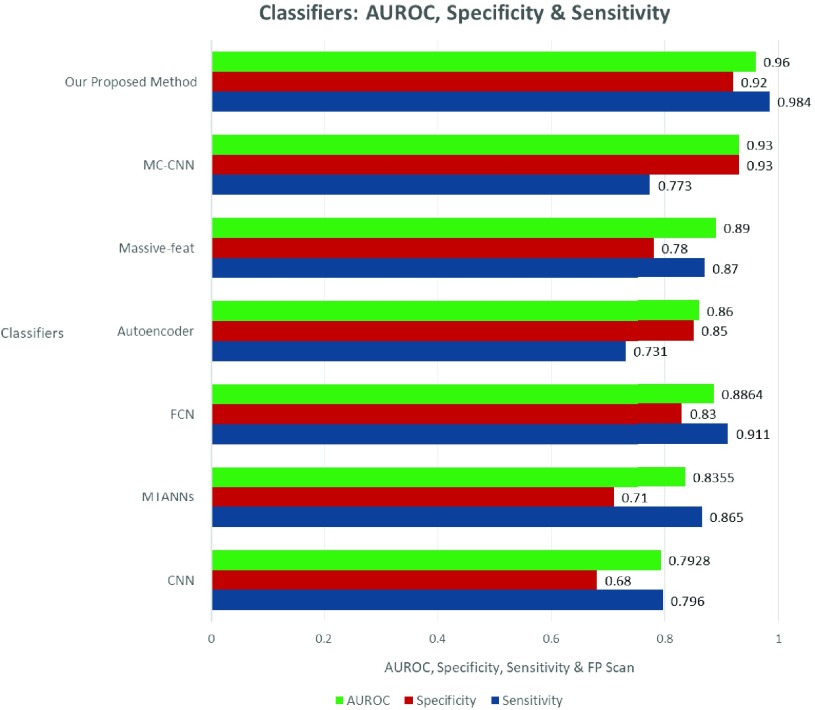
Performance comparison between our method and other existing classifiers for nodule detection on LIDC-IDR Dataset.

Performance comparison between our proposed CAD system versus state-of-the-art CAD systems on LIDC-IDR dataset is shown in Fig. 9 in terms of the average sensitivity and FPs/scan. $S$, $SP$, $FP$, $FN$, and $TP$ denotes sensitivity, specificity, false positive, false negative and true positive rate, respectively. In our experiment, if a sample with nodule is not predicted as disease in our CAD system, it is $FN$. If a sample with nodule is predicted correctly, it represents $TP$.

We calculate FP rate between the number of non-nodule samples falsely predict as nodule with a certain level of malignancy and the total amount of non-nodule samples. In the following we give the definition of FP rate:

\begin{equation*} \frac {FP}{N}=\frac {FP}{FP+TN}\tag{13}\end{equation*}

Sensitivity and specificity are calculated as:

\begin{align*} S=&\frac {TP}{TP+FN} \tag{14}\\ SP=&\frac {TN}{TN+FP}\tag{15}\end{align*}

To convert the malignancy probability output by the classifier to a binary response, we used threshold (e.g. $\tau =0.5$). However, decreasing or increasing $\tau $ will cause the classifier to produce more positive or negative predictions. We can observe in Table 1, that our proposed CAD system (3DDCNN) has attained the best performance with accuracy of 98.51%. From Table 2, we can deduce that 3DDCNN’s performance is highest in terms of sensitivity, which was found for 98.4% with a lowest FP rate of 2.1 per CT Scan among these CAD system. Our 3DDCNN performs better with mRPN than the original 3DDCNN proposed model. Fig. 8 shows the accuracy of our proposed method in comparison to the published classifiers for the nodule candidate detection. The comparison between our proposed system and the previously published CAD systems to investigate the perspectives of our 3DDCNN system was done using the average FPs/Scan as parameter shown in Table 3.

**TABLE 2:** Performance Comparison of Various Classifiers’ Sensitivity, Specificity, AUROC, FPs/Scan, and Classification Time

Classifiers	Total Cases	Total Nodules	Sensitivity	Specificity	AUROC	FPs/Scan	Time (sec)	Classes
CNN [12]	800	1738	0.796	0.68	0.7928	3.63	179.5	Malignant
MTANNs [33]	120	493	0.865	0.71	0.8355	2.62	128.6	Malignant & Benign
FCN [13]	300	893	0.911	0.83	0.8864	3.16	69.75	Malignant & Benign
Autoencoder [10]	495	1010	0.731	0.85	0.86	3.11	0.01	Malignant & Benign
Massive-Feat [9]	880	1243	0.870	0.78	0.89	3.43	32.76	Malignant & Benign
MC-CNN [14]	340	825	0.773	0.93	0.93	2.97	0.23	Malignant & Benign
Proposed 3DDCNN	1190	2361	0.984	0.92	0.96	3.39	0.011	Malignant & Benign
Proposed 3DDCNN (with mRPN)	1190	2361	0.991	0.94	0.9743	2.10	0.025	Malignant & Benign

**TABLE 3:** Performance Comparison of Our Proposed CAD System With State-of-the-Art CAD Systems to Detect and Classify Lung Cancer on LIDC Dataset

CAD Systems	Nodule Size (3mm)	Sensitivity(%)	FPs/Scan
Cascio et al. [34]	≥3	97.0%	6.1
Teramoto and Fujita [8]	5–20	80.0%	4.2
Choi and Choi [29]	3–30	95.3%	2.3
CNN [12]	≥3	81.7%	3.7
Our Proposed Method	≥3	98.4%	2.1

Fig. 6 shows true nodules (marked in green) that were missed in the traditional CNN method [12], but were detected by our proposed method, when the false positives per scan lies within the range of 1 to 4 with overall sensitivity of 0.9743. The false negatives are marked in red and are shown in the last two rows; these have similar appearance to nodules but our proposed system detected them as non-nodules using the characteristics of lung nodules obtained by our 3DDFCN, such as the example in Fig. 6 third and fourth row marked in yellow.

#### 3) Clinical Dataset

We investigated the performance of our 3DDCNN system in comparison to Cloud-Based 3DDCNN system on 120 cases from Shanghai Sixth People’s Hospital using sensitivity and average FPs/Scan as parameter shown in Table 4. Training and Testing error were obtained for both Stand-alone and Cloud-based CAD mode. With the integration of cloud computing, Cloud-Based 3DDCNN CAD system allowed the cloud server to efficiently perform the nodule detection, and enables the cloud server to reduce overall storage costs by more than 59%, while ensuring improved lung cancer detection.

**TABLE 4:** Quantitative Results Associated With Training, Testing Errors, of 3DDCNN and Cloud-Based 3DDCNN CAD System on Different Datasets

Dataset	3DDCNN CAD Mode	Training Error	Testing Error	Sensitivity	FP/scan
LIDC-IDRI	Stand-Alone	0.00448	0.00661	0.952	2.4
[22]	Cloud-Based	0.00391	0.00398	0.974	2.1
ANODE09	Stand-Alone	0.00542	0.00478	0.950	2.9
[21]	Cloud-Based	0.00419	0.00354	0.976	2.3
LUNA16	Stand-Alone	0.00391	0.00328	0.961	2.2
[20]	Cloud-Based	0.00368	0.00274	0.988	1.97
SPH6	Stand-Alone	0.00364	0.00288	0.964	2.1
Dataset	Cloud-Based	0.00294	0.00224	0.987	1.97

”SPH6” refers to Clinical Dataset from Shanghai Sixth People’s Hospital

### D. Quantitative Evaluation

As mentioned before the performance evaluation metric include Sensitivity, Specificity, AUROC, and false positives per scan (FPs/Scan). For further quantitative evaluation of our proposed work with the existing RCNN based methods, we used the statistical performance evaluation based on error calculation metrics which were used to determine the error rate on the testing dataset i.e. Clinical Dataset (120 cases from Shanghai Sixth People’s Hospital). To calculate the average difference between the radiologists nodule detection and the proposed method nodule detection we proposed the Detection Error Rate (${e}_{D}$). The Detection Error Rate (${e}_{D}$) was calculated as:

\begin{equation*} e_{D}({I}_\phi) = \sum _{i=0,k} e_{I}(G_{k}(x_{i}),h \mid {I}_\phi)\tag{16}\end{equation*}

where $e_{D}({I}_\phi)$ is metric that was used to calculate the error rate of detection phase, $k$ represents the number of nodules, $\phi $ represents nodule in Image $I$, $h$ is the score derived for each layer of proposed model, $x_{i}$ is the real value of the resultant metric for each layer, and $G$ is the mean value for outputs delivered from previous layer. Another metric was proposed to calculate the average error between the nodule classification by doctors from Shanghai Sixth People’s Hospital and the proposed methods classification. The metric, Classification Error Rate (${e}_{C}$) can be defined as:

\begin{equation*} {e}_{C} = \frac {1}{2}\log \frac {1-e_{D}({I}_\phi) }{e_{D}({I}_\phi) }\tag{17}\end{equation*}

Since, we obtained the detection results on three malignancy levels $m$, we used 10-fold cross-validation to merge the detection results of three levels. The performance of nodule classification was validated using the LIDC dataset and the LUNA16’s dataset distribution criteria of 10-fold cross-validation of patient-data. Owing to the minor differences among the malignant and the benign nodules, 900 epochs were used for the various learning rates [0.001,0.001,0.0001]. Detailed step-by-step performance of our proposed method is provided in the Table 5. We used mean Average Precision also refer to as $AP$ for the detection phase evaluation. Table 5 represents the AP metric which averages APs across IoU thresholds from 0.5 to 0.95 with an interval of 0.05. For our proposed model AP, we took three different IoU thresholds referring to three AP i.e. AP_50_, AP_75_ and AP_*m*_.

**TABLE 5:** Quantitative Results for 3DDCNN in Terms of Mean IoU and Average Precision Against 3DDCNN Network Layers. Three AP are Considered (AP_50_, AP_75_ and AP_m_ at Different IoU Thresholds) Were Selected Showing Mean IoU Values of 3DDCNN Layers $i$ to $n$

Method	Layer	Testing Dataset			
		mean IoU		Average Precision (AP)	
		mIoU	AP_m_	AP_50_	AP_75_
3DDCNN	$Layer_{i}$	37.7	49.4	39.5	34.8
	$Layer_{j}$	48.7	47.4	35.2	36.2
	$Layer_{k}$	33.4	48.9	51.1	32.3
	$Layer_{n}$	42.5	50.8	50.7	37.5
3DDCNN Using mRPN	$Layer_{i}$	40.3	36.5	54.2	33.9
	$Layer_{j}$	37.1	52.4	48.2	35.7
	$Layer_{k}$	42.0	42.6	48.9	47.2
	$Layer_{n}$	45.8	53.5	51.6	44.2

To quantitatively evaluate the results for our proposed method, we have measured Mean Detection Error Rate (Mean ${e}_{D}$), Mean Classification Error Rate (Mean ${e}_{C}$), Variance Detection Error Rate (Var ${e}_{D}$), Variance Classification Error Rate (Var ${e}_{C}$), Standard Deviation Detection Error Rate (Std ${e}_{D}$), Standard Deviation Classification Error Rate (Std ${e}_{C}$), Mean Average Precision (m-AP)and Processing-time (Time) of the CT images in the testing set of our clinical dataset with Mask R-CNN [35], RetinaNet [36], Retina U-Net [37], Fast R-CNN [38], Faster R-CNN [24] techniques. It can be seen in Table 6 that, our proposed method achieved comparatively good results for Detection and Classification than the state-of-the-art.

**TABLE 6:** Comparison of Proposed 3DDCNN With State-of-the-Art on Clinical Dataset Using Different Statistical Metrics, Namely, Mean Detection Error Rate (Mean ${e}_{D}$), Mean Classification Error Rate (Mean ${e}_{C}$), Variance Detection Error Rate (Var ${e}_{D}$), Variance Classification Error Rate (Var ${e}_{C}$), Standard Deviation Detection Error Rate (Std ${e}_{D}$), Standard Deviation Classification Error Rate (Std ${e}_{C}$), Mean Average Precision (m-AP) and Processing-Time (Time)

Methods	Score	Mean ${e}_{D}$	Mean ${e}_{C}$	Var ${e}_{D}$	Var ${e}_{C}$	Std ${e}_{D}$	Std ${e}_{C}$	m-AP (%)	Time (min)
Mask R-CNN [35]	83.63±1.34	2.20±1.2	2.38±4.5	2.3±0.5	1.23±0.27	4.18±3.30	4.3±1.8	54.3±2.5	11
RetinaNet [36]	83.68±4.10	3.94±0.71	1.93±2.8	4.6±1.2	1.62±0.30	3.40±4.31	3.9±3.0	56.4±2.8	7–8
Retina U-Net [37]	89.4±.91	2.64±2.3	1.84±2.3	1.1±0.7	1.79±0.95	2.82±2.32	4.2±1.7	62.9±3.4	5–6
Fast R-CNN [38]	79.5±3.70	5.69±3.94	−0.04±4.4	5.8±1.3	1.97±0.90	8.58±8.02	9.9±2.2	44.9±3.0	21.7
Faster R-CNN [24]	76.3±2.31	6.07±2.92	1.04±6.8	1.7±0.6	1.61±0.99	7.41±4.31	9.31±5.1	52.8±10.7	17.2
Proposed 3DDCNN	84.2±1.20	3.32±2.8	2.96±1.3	1.2±2.35	1.24±0.51	3.69±3.87	4.01±5.2	55.8±4.1	4–6
Proposed 3DDCNN (Using mRPN)	87.8±1.35	3.30±0.58	1.28±0.19	1.4±2.48	1.10±0.26	2.88±3.61	4.5±3.8	59.7±3.2	2–4

Table 6 presents the scoring criteria: statistical parameters such as mean, variance, standard deviation which are used for both types of error rates i.e. Detection error rate and Classification error rate. Mean Average Precision m-AP as 100% indicate perfect detection, the metric $e_{D}({I}_\phi)$ represents the error rate between the radiologists from Shanghai Sixth People’s Hospital nodule detection and the detection done by our proposed method and the term ${e}_{C}$ represents the nodule classification by doctors from Shanghai Sixth People’s Hospital and our proposed methods classification. Computed results are presented in Table 6. Usually, it is difficult to compare the proposed study with state-of-the-art due to diverse datasets and different evaluation metrics. However, in our case, we compared our proposed work with other deep learning based methods which were designed for object detection but performed well for nodule detection. The evaluation results of Fast R-CNN [38] and Faster R-CNN [24] are costly due to the rigorous object detection process, they are not specifically designed for the lung nodules, therefore, their detection error rate is comparatively higher than the rest of the methods. On the other hand, RetinaNet [36], Mask R-CNN [35] and Retina U-Net [37] performed better than the Fast R-CNN and Faster R-CNN. Although the assessment is conducted on the clinical dataset, Table 6 still emphasizes 3DDCNN advantages in terms of computational duration (of around 219±25.47 seconds) over other methods.

### E. Qualitative Evaluation

**FIGURE 10.**
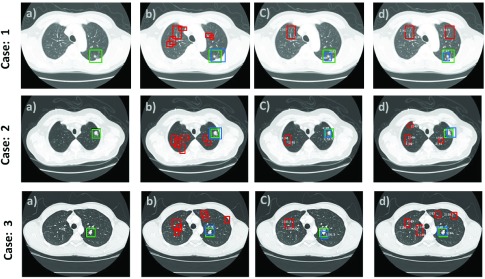
Qualitative results from the performance comparison between our CAD system versus state-of-the-art CAD system on Clinical Dataset having a confidence threshold of 95%. Green represents the Ground truth box. Red as predicted by Retina U-Net [37] and blue represents our proposed model predictions. Three random cases out of 120 cases from Shanghai Sixth People’s Hospital are selected. Left-most images (a) show the ground truth of lung CT scan, images (b) in all cases show the box-predictions of Retina U-Net (2 foreground classes) as well as the prediction of our proposed 3DDCNN model, images (c) represent the remaining predictions after prediction of nodule along with some False Positive predictions, images (d) After the False Positives reduction step, leaving the correct prediction with confidence scores as high as 87%.

Qualitative results from Fig. 10 show that our proposed model 3DDCNN performed well in most of the clinical dataset cases (total 120 cases) from Shanghai Sixth People’s Hospital while the state-of-the-art Retina U-Net [37] method outperformed our proposed method in some of the CT-scan from clinical dataset. We randomly selected 3 cases for our qualitative analysis. The step-by-step evaluation of the proposed work with Retina U-Net in the set of four visualizations of the central CT-scan slices for ground-truth and the classification accuracy is shown in the form of set of images (a), (b), (c), and (d). Images marked as (a) in each case (left-most images in each row) represents the ground-truth of given CT scan. Images (b) in each case depicts the box-prediction of the Retina U-Net (red boxes) while the prediction of our proposed method is represented with blue boxes. There are minor deviations of the markings of our proposed model (shown in blue) from the ground truth (shown in green) as shown in images (c). The last images (d) in each row shows the final results after the FP reduction, resulting in the correct prediction with approximately 87% confidence score. The proposed 3DDCNN performs well for nodule detection whereas the Retina U-Net performs better in case of nodule classification by achieving higher classification accuracy. Qualitative validation of our proposed 3DDCNN model against the annotations demonstrated that the detection accuracy of our proposed 3DDCNN performed equally well as the radiologists while in some cases even better than the radiologists annotations.

## V. Conclusion

For the detection of lung cancer, CAD systems are developed to assist the radiologist in the process of nodule detection by providing a reference opinion. From the performance comparisons it is evident that our proposed model 3DDCNN attained the highest results against other state-of-the-art systems for sensitivity and FPs per scan. Although the current tested performance metric of 3DDCNN is relatively high, it could be further improved. The performance was relatively less accurate in detecting micro nodules, therefore future work will investigate the detection of micro nodules whose diameter is less than 3 mm. To ensure our solution is scalable, future work will consider extending the training stage to include data from hospitals worldwide. Integrating more data-augmentation methods to increase the training sample in order to achieve more robustness and reduce the overfitting problem brought by local optimal. Another future direction for lung cancer CAD system is to propose CAD system that performs well on all nodule types maintaining good performance in terms of sensitivity and FPs/Scan, even if the dataset contains relatively less amount of such nodule types samples.
